# Genetic instability is prevented by Mrc1-dependent spatio-temporal separation of replicative and repair activities of homologous recombination: Homologous recombination tolerates replicative stress by Mrc1-regulated replication and repair activities operating at S and G2 in distinct subnuclear compartments

**DOI:** 10.1002/bies.201300161

**Published:** 2014-02-26

**Authors:** Félix Prado

**Affiliations:** Departamento de Biología Molecular, Centro Andaluz de Biología Molecular y Medicina Regenerativa (CABIMER), Consejo Superior de Investigaciones Científicas (CSIC)Seville, Spain

**Keywords:** DNA checkpoints, homologous recombination, Rad51, repair centers, replication

## Abstract

Homologous recombination (HR) is required to protect and restart stressed replication forks. Paradoxically, the Mrc1 branch of the S phase checkpoints, which is activated by replicative stress, prevents HR repair at breaks and arrested forks. Indeed, the mechanisms underlying HR can threaten genome integrity if not properly regulated. Thus, understanding how cells avoid genetic instability associated with replicative stress, a hallmark of cancer, is still a challenge. Here I discuss recent results that support a model by which HR responds to replication stress through replicative and repair activities that operate at different stages of the cell cycle (S and G2, respectively) and in distinct subnuclear structures. Remarkably, the replication checkpoint appears to control this scenario by inhibiting the assembly of HR repair centers at stressed forks during S phase, thereby avoiding genetic instability.

## Introduction

Faithful replication of the complete genome is essential for preventing any loss of genetic information. However, this is not an easy task; in fact, the genetic instability that accompanies tumor progression during early stages is associated with replicative stress 1–3. A high risk of mutations and genome rearrangements during DNA replication is linked to the replication fork, a highly dynamic nucleosome-free structure with DNA ends and stretches of single-stranded DNA (ssDNA) susceptible to being the substrate of nucleases and DNA processing enzymes. This fragile structure has to deal with a number of obstacles that hamper its advance, such as DNA adducts generated by endogenous and exogenous agents, abasic sites generated spontaneously or by processing damaged bases, incorporation of rNTPs instead of dNTPs, DNA-binding proteins, specific DNA structures (such as G-quadruplexes, hairpins, and DNA/RNA hybrids), compacted chromatin structures, and even other DNA processes like transcription 4–7. Additionally, unbalanced supplies of dNTPs or histones strongly threaten fork stability 8–10. Not surprisingly, cells are endowed with different mechanisms to protect, repair, and restart stressed replication forks. Of these, homologous recombination (HR) plays an essential role, and mutations in genes that encode recombination proteins are associated with defective proliferation, genomic instability, and tumorigenesis 11. Unexpectedly, though, numerous data have shown that the DNA replication checkpoint inhibits HR repair at double-strand breaks (DSBs) and stressed forks 12,13. HR inhibition during S phase is in apparent contradiction with the requirement of recombination proteins to protect and repair stressed forks. In light of recent results, I propose that HR responds to replication stress through replicative and repair activities that are temporarily separated via Mrc1-dependent inhibition of the repair HR centers during S phase. In this way, the DNA replication checkpoint would prevent unscheduled and mutagenic repair events caused by the assembly of repair centers at stressed replication forks. In addition to resolving the aforementioned paradox, this model provides a new scenario to understand why HR is essential in maintaining genome integrity under replicative stress.

## Homologous recombination proteins promote the repair of stalled and broken replication forks

The best way to fix DNA is to use an undamaged template, which is the rationale behind HR. The mechanisms of HR use intact homologous DNA molecules to repair DNA breaks, thus preserving genetic information. This is achieved by invading the donor molecule with the 3′-end of the broken molecule. HR has been most extensively studied during DSB repair 14, in which the 5′-end of the broken molecule is resected, generating a 3′-ended ssDNA fragment that is then coated by the ssDNA binding complex RPA (Box 1). The mediator proteins Rad52 and BRCA2 (see Table1 for yeast and vertebrate orthologs involved in HR and S-phase checkpoints) compete with RPA to load Rad51 (RecA in bacteria), forming a nucleofilament that invades a homologous sequence. This invasion step generates a D-loop structure that is further stabilized by DNA synthesis and Rad51-mediated strand exchange, events that can lead to gene conversion if the template is not identical. After this critical step, a recombination event can occur by different mechanisms that may or may not involve reciprocal exchange of DNA between the two molecules (crossover) (Box 1).

**Box 1**

**Mechanisms of DSB-induced homologous recombination**

DSB repair by HR is initiated by resecting the 5′-ends of the break to generate the 3′-ended ssDNA molecules, which are common intermediates in all recombination processes. DNA resection is initiated by the Mre11-Rad50-Xrs2/Nbs1 (MRX/N) complex and Sae2/CtIP that, together with BRCA1, counteract the inhibitory activities of the NHEJ Ku complex and the checkpoint protein 53BP1. Then, the nuclease Exo1 and the helicase–nuclease Sgs1–Dna2 extend DNA resection.

The mediator proteins Rad52 and BRCA2 compete with the ssDNA binding complex RPA to load Rad51 and form a nucleofilament involved in the search and invasion of a homologous sequence. This invasion leads to the formation of a D-loop structure that is further enlarged by DNA synthesis and Rad51-mediated strand exchange. Once this recombination intermediate is formed, HR can proceed through different mechanisms.

(1) Synthesis-dependent strand annealing (SDSA). The strand exchange intermediate is reverted, and the newly synthesized molecule reannealed with the other 3′-ended ssDNA molecule. SDSA can also occur after the second 3′-ended ssDNA molecule has been captured by the D-loop; in any case, the product is a non-crossover.
(2) Double-strand break repair (DSBR). The D-loop reaches the resected 5′-ends to generate two Holliday junctions (HJ). These can be dissolved by a helicase–topoisomerase complex (leading to non-crossover) or resolved by specific nucleases (leading to either non-crossover or crossover, depending on whether the HJs are cleaved in the same or opposite orientation).
(3) Break-induced replication (BIR). The second 3′-ended ssDNA is not captured, while the invading 3′-end primes DNA replication through conservative DNA synthesis.
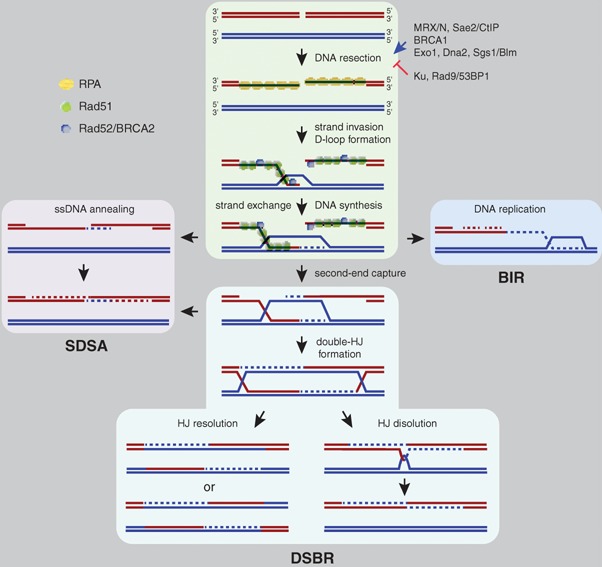

**Table 1:** Proteins involved in homologous recombination and S phase checkpoints^a^

*S. cerevisiae*	*S. pombe*	Vertebrates	Function
Rad51	Rhp51	Rad51	DNA strand exchange
Rad52	Rad22	Rad52	Rad51-ssDNA assembly
		BRCA2	Rad51-ssDNA assembly
RPA	RPA	RPA	ssDNA binding
Mre11	Rad32	Mre11	DNA resection
Rad50	Rad50	Rad50	DNA resection
Xrs2	Nbs1	Nbs1	DNA resection
Sae2	Ctp1	CtP1	DNA resection
		BRCA1	DNA resection
Exo1	Exo1	Exo1	DNA resection
Dna2	Dna2	Dna2	DNA resection
Sgs1	Rqh1	RECQ1	Fork reversal/regression
		BLM	Fork reversal/regression
		SMARCAL1	Fork reversal/regression
Mus81	Mus81	Mus81	Fork/HJ-like structure cleavage
Mec1	Rad3	ATR	Checkpoint sensor
Tel1	Tel1	ATM	Checkpoint sensor
Rad9	Crb2	53BP1	DNA damage checkpoint mediator
Mrc1	Mrc1	Claspin	DNA replication checkpoint mediator
Chk1	Chk1	Chk1	Checkpoint effector
Rad53	Cds1	Chk2	Checkpoint effector

a Only the proteins – orthologs or functional counterparts – and functions mentioned in the text are shown. *S. cerevisiae*, *Saccharomyces cerevisiae*; *S. pombe*, *Schizosaccharomyces pombe*.

**Figure 1:**
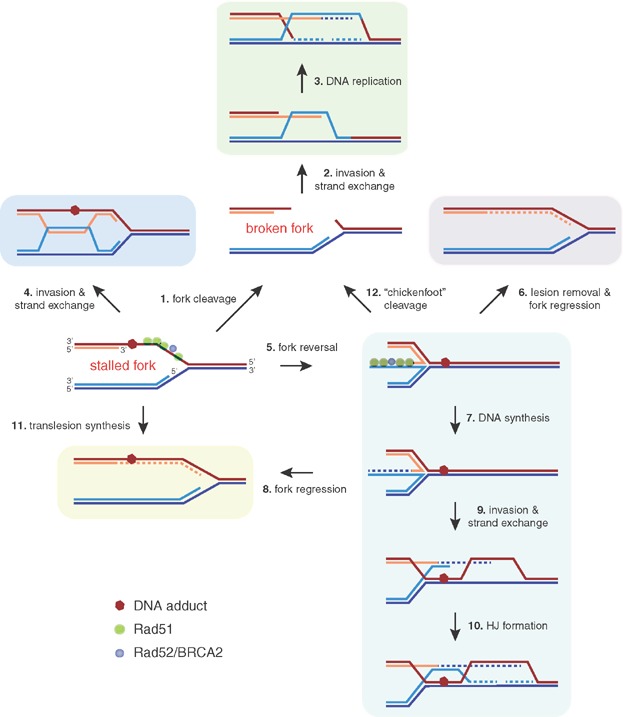
Mechanisms of replication fork restart by homologous recombination. Replication forks can stall or break under conditions that impair their normal progression. At stalled forks, the 3′-ended nascent strand might invade the sister chromatid to generate either an HJ-like structure to bypass the blocking lesion (*step 4*) or a “chickenfoot” structure by fork reversal (*step 5*). Fork reversal would allow the replication-blocking lesion to be repaired, the lesion to be bypassed by DNA synthesis and regression, or the fork to be restarted downstream of the lesion by strand invasion and double HJ (*steps 6*, *7*–*8*, and *9*–*10*, respectively). Apart from these fork stalling and template switching (FoSTeS) mechanisms, Rad51 might bypass the blocking lesion by recruiting translesion-synthesis polymerases able to incorporate dNTPs opposite DNA adducts (*step 11*). An alternative but not mutually exclusive mechanism might facilitate fork restart by preventing nuclease-mediated degradation of stressed forks (see forks coated with Rad52/BRCA2 and Rad51). At broken forks, the one-ended DSB generated by fork breakage might be resected and invade the sister chromatid, generating a HJ behind the reassembled replisome (*steps 1*–*3*). A similar BIR-like mechanism would be required if the “chickenfoot” structure generated upon fork stalling is cleaved by specific endonucleases (*step 12*). Dashed lines indicate newly synthesized DNA.

The breakage of stressed replication forks is an important source of DSBs. HR might wait for the oncoming fork to generate a two-ended DSB or, alternatively, the one-ended DSB generated at fork breakage can be used to restart replication (Fig. 1, steps 1–3). HR-dependent restart of broken forks has been well established in bacteria 15 and seems to also operate in eukaryotes 9,16–18, for which it is clear that cells are able to initiate replication from an induced DSB (break-induced replication, BIR) 19 (Box 1). In BIR, the 3′-ended molecule can invade a homologous DNA sequence, located either in an allelic or an ectopic position, and prime DNA synthesis for hundreds of kilobases. This occurs through a mechanism that requires all essential DNA replication factors – DNA polymerases and helicases – except the origin recognition complex (ORC) and the kinase Cdc6, which are required to assemble a pre-replication complex 20. It is therefore possible that a similar mechanism might operate for the restart of broken forks using the sister chromatid as a template.

DSBs are not the only source of recombinogenic DNA damage. In fact, most spontaneous HR events are initiated by non-DSBs DNA lesions 21 that are likely associated with replicative problems 22. These problems can be due to a reduction in the available pools of dNTPs or defects in DNA synthesis processivity, which can be induced with drugs like hydroxyurea or aphidicolin, respectively. Alternatively, replication forks can stall or collapse – the replisome is disassembled – without breakage when they encounter DNA blocks (e.g. UV light-induced DNA photoproducts, alkylated bases, abasic sites, or DNA-binding proteins), which uncouple the processes of DNA unwinding and synthesis. These situations lead to an accumulation of ssDNA at the fork, which trigger different mechanisms to overcome the problem. These mechanisms, which have been studied in detail in response to UV light and alkylating agents like methyl-methane sulfonate (MMS), facilitate the replication fork restart downstream of the lesion and to fill in the ssDNA gaps, prioritizing DNA replication over the repair of the initial lesion 23. For this reason, they are referred to as DNA damage tolerance mechanisms. HR proteins play essential roles both in response to replication inhibitors and in the DNA damage tolerance response by processes that remain unclear. In mammalian cells, Rad51 and BRCA2 are essential for replication forks to advance in both unperturbed and stressed conditions 24,25. In yeast, Rad51 and Rad52 are dispensable for normal replication but are required for replication fork progression through DNA adducts 12,26,27, and cells accumulate ssDNA fragments at the forks in their absence 28,29. Thus far, different mechanisms have been proposed to explain how HR can promote the stabilization and advance of stressed forks. The invasion and strand-exchange activities of Rad51 might bypass a lesion by invading the intact sister chromatid (Fig. 1, step 4), as suggested by the accumulation of Rad51-dependent HJ-like structures in yeast mutants that are defective in HJ dissolution 30. Likewise, Rad51 might promote fork reversal by annealing the nascent strands (Fig. 1, step 5), as shown for RecA in vivo and for human Rad51 in vitro 31,32. Additionally, fork reversal can be promoted by helicases (e.g. RecQ and SMARCAL1) 33,34. In any case, fork reversal would allow the replication-blocking lesion to be repaired, the lesion to be bypassed by DNA synthesis and regression, or the fork to be restarted downstream of the lesion by strand invasion and HJ formation (Fig. 1, steps 6, 7–8, and 9–10, respectively). As an alternative to these fork stalling and template switching (FoSTeS) models, Rad51 might also bypass the obstacle by recruiting translesion-synthesis polymerases able to incorporate dNTPs opposite DNA adducts, as supported by the physical and functional interactions between human Rad51 and Polη under conditions of replicative stress 35,36 (Fig. 1, step 11). In addition, human Rad51 is required to restart replication after hydroxyurea-mediated dNTPs depletion by inhibiting the nuclease activity of Mre11 24, similar to the nuclease-inhibitory activity proposed for RecFOR in bacteria 37. These results suggest a scenario in which recombination proteins facilitate replication by protecting the fork. Finally, the “chickenfoot” structure generated by fork reversal could also be cleaved by specific endonucleases to generate a broken fork 38 that would be repaired by BIR-like mechanisms (Fig. 1, step 12).

## Homologous recombination proteins escort replication forks

Regardless of the mechanism, recombination proteins might be directly targeted to stressed forks, or they might travel with unperturbed forks to act once they encounter the obstacle. Using a molecular approach to directly follow the binding of recombination proteins to the fork, my group has recently shown in *S. cerevisiae* that Rad52 and Rad51 bind to unperturbed forks, and that this interaction is not increased by DNA damage 27. Since none of these proteins is required for DNA replication under unperturbed conditions in yeast 26, we proposed that Rad52 and Rad51 escort the fork to promote DNA replication through damaged DNA 27. Supporting the idea that this escort function is conserved, Rad51 has been shown to be associated with unperturbed replicating chromatin in human cell lines and in *Xenopus* egg extracts 17,29,39, and the absence of Rad51 in yeast and *Xenopus* leads to an accumulation of ssDNA at the fork that is independent of exogenous DNA damage 29. In fact, different proteomic approaches using human cell lines aimed at isolating factors enriched at or in the proximity of replication forks have recently revealed the presence of the recombination proteins Mre11 and Rad50 40,41. Apart from these components of the core recombination machinery, other factors involved in processing the fork that likely collaborate with HR have been found to travel with the fork in unperturbed conditions. This is the case for the helicase SMARCAL1, which colocalizes with replication factories and helps rescue stalled forks by promoting fork reversal and regression 42. Notably, homologous recombination proteins are not the only repair factors that travel with the fork; other replication fork escorts reported so far include the mismatch repair proteins Msh2, Msh3, and Msh6 40,43, the DSB repair proteins Ku70 and Ku80 41,44, and the single-stranded DNA repair protein PARP1 44.

## Homologous recombination as a source of replication-associated genetic instability

Using a homologous DNA sequence as an information donor for lesion repair makes HR a “safer” fork restart mechanism than other processes. In the case of DSBs, an alternative pathway is non-homologous end joining (NHEJ), where the two ends of the broken molecules are ligated 45. However, NHEJ usually requires processing of the ends and, consequently, causes a loss of genetic information. Additionally, NHEJ cannot deal with one-ended DSBs generated by breakage of the replication fork. Likewise, template switching by HR provides an error-free mechanism of DNA damage tolerance as compared with translesion synthesis (TLS), an error-prone process in which the DNA lesion that hampers fork progression is bypassed by incorporating a nucleotide opposite to the lesion 46.

However, despite the fact that it is essential for the restart of stressed and broken forks, HR involves a risk for genome integrity. First, DNA synthesis during both two-ended DSB-induced HR and BIR is highly mutagenic as compared with normal DNA replication (1,000–2,000 times higher) 47,48. In the case of BIR, this inaccuracy is associated with an accumulation of ssDNA behind a migrating bubble that drives conservative DNA synthesis 49–51. Therefore, fork restart by replisome reassembly may represent a safer mechanism than BIR-like processes.

Second, HR repair involves the formation of replication intermediates and intermolecular junctions that have to be properly resolved in order to prevent genetic instability. In the absence of activities involved in the formation and resolution of recombination intermediates (e.g. the RecQ helicases or the nuclease Mus81), unrestrained HR causes genome rearrangements 22,52,53.

**Figure 2:**
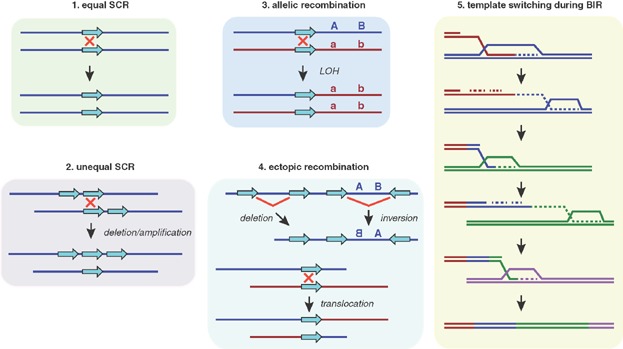
Mechanisms of homologous recombination-mediated genome rearrangements. Unless the sister sequence is used as the template (*equal SCR* (1), equal sister-chromatid recombination), repairing recombinogenic DNA lesions can generate genetic instability. Examples of genome rearrangements associated with crossovers include: *unequal SCR* (2), which leads to deletions and amplifications when it occurs between direct repeats, and acentric and dicentric chromosomes when it occurs between inverted repeats (not shown); *allelic recombination* (3) – between homologous chromosomes – which leads to loss of heterozygosity (LOH); and *ectopic recombination* (4) between repeats located either in different or the same chromosomes in a non-allelic position, which leads to deletions, inversions, and translocations, depending on the position of the repeats. Additional genetic instability can arise by gene conversions as well as one-ended template switching events (such as shown in Fig. 1) through unequal, allelic, or ectopic HR (not shown). Finally, multiple rounds of strand invasion, DNA synthesis, and dissociation during BIR can lead to a particularly catastrophic event if they occur between dispersed DNA sequences (*template switching during BIR* (5)). Dashed lines indicate newly synthesized DNA.

Third, HR is a potential source of genetic instability when it does not go through an equal sister-chromatid recombination event. It can lead to loss of heterozygosity, an event that can uncover deleterious mutations. In addition, HR can generate copy number variations (CNV) – amplifications and deletions – as well as inversions and translocations when the information donor is an ectopic DNA repeat located in the same chromosome, a non-homologous chromosome, or even the sister chromatid (Fig. 2). Even though these chromosomal rearrangements can occur through two-ended DSB repair mechanisms, they are expected to mainly occur by ectopic BIR and FoSTeS processes during replicative stress. In fact, BIR and FoSTeS seem to account for some of the CNVs and complex genomic rearrangements associated with cancer and genetic disorders 54–57. Two major features of BIR, which might be formally extended to FoSTeS, can explain their highly deleterious effects on genome integrity. First, BIR can proceed several rounds of strand invasion, DNA synthesis, and dissociation, and this can lead to gross chromosome rearrangements if it occurs within dispersed repeat sequences 58 (Fig. 2). Second, BIR can proceed through a non-homologous recombination mechanism by invading microhomologous DNA sequences (MMBIR, microhomology-mediated BIR) 59, which might explain the presence of microhomologies at the breakpoints of complex genomic rearrangements that have been associated with genetic diseases and cancer 54,60.

Studies in yeast have shown that although these events are rare compared to equal sister-chromatid recombination events 61,62, their frequency is high enough to threaten genome integrity 63, especially if stimulated by agents that impair replication fork advance. In these cases, fork restart can proceed by template switching events using ectopic DNA repeats 64. In conclusion, HR has to be tightly regulated to avoid unscheduled recombination events associated with the restart of stressed replication forks, as these events can lead to a dramatic loss of genetic information.

## An unresolved paradox: Replication checkpoints inhibit recombinational repair, yet recombination is required to restart stressed forks

How do cells control HR to promote fork protection/restart and to prevent unscheduled and deleterious recombination events? A key mechanism in this regulation is the DNA damage checkpoint, which operates throughout the cell cycle to prevent chromosome duplication or segregation until damaged DNA is repaired. A number of sensor proteins detect different types of DNA lesions, thereby triggering a phosphorylation-mediated signal cascade that then activates a plethora of responses to coordinate the DNA damage repair/tolerance with cell cycle progression 65. The DNA damage checkpoint works together with cyclin-dependent kinases (CDKs), master regulators of cell cycle progression, to avoid HR-mediated genetic instability through post-translational modifications of recombination proteins. These modifications prevent unrestrained recombination by regulating the stability and reversibility of critical recombination intermediates (DNA resection, Rad51/ssDNA formation, strand invasion and D-loop formation, and joint molecules resolution) through pro- and antirecombinogenic functions 66.

A key step in the choice of the repair mechanism is DNA end resection, which is tightly regulated during the cell cycle by the CDKs and the DNA damage checkpoint. DNA resection prevents NHEJ and promotes HR 67, but extensive resection inhibits BIR 68. The Mre11 complex and Sae2/CtIP counteract the DNA resection inhibitory activity of the Ku complex 69, and initiate DNA resection 70–72. This produces the substrate for further nucleolytic degradation, which occurs in two parallel pathways driven by the nuclease Exo1 and the helicase–nuclease complex formed by Sgs1/Blm and Dna2 73–75. CDK-dependent phosphorylation of Sae2/CtIP and Dna2 is required for proper resection 76–78, thus restricting HR to the S and G2 phases of the cell cycle. Additionally, CtIP phosphorylation promotes its interaction with the tumor suppressor protein BRCA1, which is required to recruit CtIP to the break and for further DNA end resection 79. In contrast, the DNA damage checkpoint protein Rad9/53BP1 negatively regulates DNA resection, the latter by counteracting the resection activities of CtIP and BRCA1 80. Since checkpoint activation is triggered by an accumulation of ssDNA 81, this strategy leads to a tight control of the tract of DNA resection, and consequently of the repair mechanism.

In addition to the DNA damage checkpoint, cells are endowed with a DNA replication checkpoint that operates only during the S phase as it is specifically activated by stressed replication forks 82,83. The DNA damage checkpoint and the DNA replication checkpoint are highly interconnected networks that share many signals, components, and responses. Still, they can be genetically distinguished by the mediator protein that connects the two major sensors (Mec1/Rad3/ATR and Tel1/ATM) with the two major effectors (Rad53/Cds1/Chk2 and Chk1) of both pathways: Rad9/Crb2/53BP1 is present in the DNA damage checkpoint, and Mrc1/Claspin in the DNA replication checkpoint 84–88 (Table1). Mrc1/Claspin travels with the fork and ensures that DNA synthesis and DNA unwinding processes are coupled under unperturbed conditions through a replicative, checkpoint-independent activity; when the forks are stressed, Mrc1/Claspin promotes checkpoint activation by hyper-phosphorylation of the effectors; finally, the Mrc1 replicative activity facilitates fork restart upon block release 85,89–91. However, Rad9 can bind to damaged forks in *mrc1* yeast mutants 91, which explains the backup checkpoint activity of Rad9 in the absence of Mrc1 86. Besides being involved in general checkpoint responses like inhibition of mitosis and activation of DNA repair mechanisms, the DNA replication checkpoint is specifically aimed at stabilizing stressed replication forks 92,93. For instance, checkpoint activation by dNTPs depletion in yeast and mammalian cells phosphorylates the nucleases Exo1 and Mus81 and inhibits replication fork cleavage and genetic instability 94–96. In mammalian cells, the inhibition of Mus81 and other endonucleases that can process aberrant replication forks is at least in part mediated by ATR-dependent phosphorylation of the fork escort SMARCAL1, which modulates its fork regression activity 97,98.

Intriguingly, experiments in yeast have shown that a major task of the DNA replication checkpoint is to inhibit HR repair 99. An important and conserved feature of HR repair occurs through its association with subnuclear foci 100. HR foci can be detected after treating cells with agents that induce DSBs and therefore activate the DNA damage checkpoint 101–104. However, additional activation of the DNA replication checkpoint with drugs that interfere with proper fork dynamics, like MMS or hydroxyurea, suppresses the formation of DSBs-induced HR foci 12,105. At the molecular level, hydroxyurea or MMS activation of Mrc1 inhibits DNA resection and thereby the formation of the substrate for Rad52 and Rad51 12.

The DNA replication checkpoint inhibits not only HR at DSBs but also at forks stressed by dNTPs depletion. Yeast cells treated with hydroxyurea do not form HR foci 102; intriguingly, studies in *S. pombe* have shown that the formation of HR foci upon hydroxyurea treatment requires cells to complete replication, unless they are checkpoint deficient and thus form persistent HR foci in S phase. At the molecular level, the DNA replication checkpoint prevents the formation of aberrant recombination-dependent structures at the fork 13. These data suggest that the DNA replication checkpoint promotes a temporal separation of replication and HR by restricting the latter to G2. Accordingly, avian DT40 cells do not form HR repair centers in response to replication-associated DNA lesions until G2 106. However, HR inhibition during S phase is in apparent contradiction with the requirement of recombination proteins to protect and help advance stressed forks.

## Spatio-temporal regulation of replicative and repair recombination activities

Intriguingly, fork restart after dNTPs depletion in mammalian cells requires Rad51 and BRCA2 but not their repair activity 24. In fact, human Rad51-mediated restart after a short hydroxyurea treatment is not associated with the formation of HR repair centers, while forks collapsed after a long hydroxyurea treatment do not restart but are repaired at HR repair centers 39. Both roles of Rad51 in response to replicative stress require BRCA1 and CtIP, which regulate the amount of phosphorylated RPA2 in the case of stalled fork restart, suggesting that BRCA1/CtIP promotes Rad51 recruitment by modulating DNA resection 107. These results support a role for the tumor suppressor genes BRCA1 and BRCA2 in HR-mediated restart of stalled replication forks.

**Figure 3:**
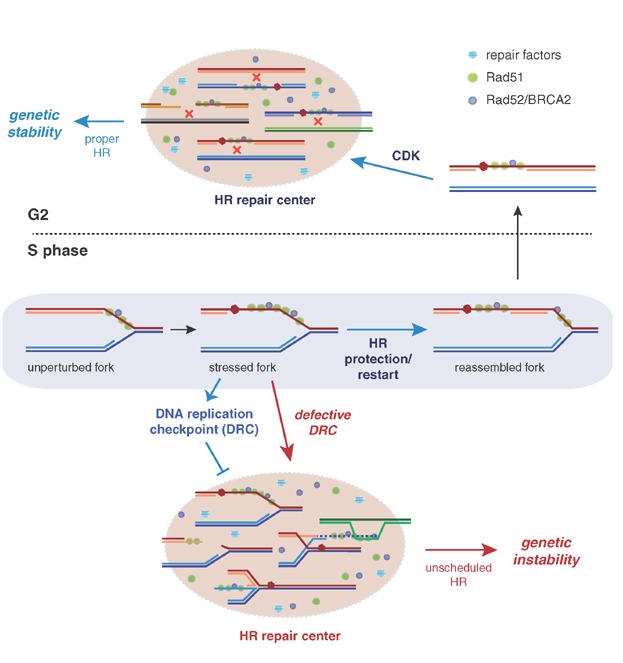
Model for the cell cycle control of recombinational replicative and repair functions by the DNA replication checkpoint. Rad52 and Rad51 bind to unperturbed replication forks. Under conditions that impair fork progression, Rad51 and Rad52 promote the protection/restart of the stressed fork through the reassembly of a canonical replisome (see putative mechanisms in Fig. 1). ssDNA accumulation at stressed forks activates the DNA replication checkpoint (DRC), which prevents the formation of DNA repair centers. Once replication is completed, DRC is switched off, repair centers are assembled through a mechanism that requires the kinase activity of CDK, and recombinogenic lesions are properly repaired. In mutants defective for DRC, the HR repair centers might prematurely assemble at stressed replication forks, thereby interfering with proper DNA replication and promoting genome rearrangements by unscheduled recombination (see putative mechanisms in Fig. 2).

This dual role of HR in replication and repair is not limited to the hydroxyurea treatment condition in mammalian cells. Thus, although yeast Rad52 and Rad51 are detected at MMS-induced DNA lesions not only in S phase but also when replication is largely completed, Rad52 and Rad51 foci do not form until G2. Therefore, lesion repair is not coupled to the fork but in fact prevented during S phase 27. Since yeast Rad52 and Rad51 are bound to the forks and are required for their advance through alkylated DNA 12,26,27, these results indicate that Rad52 and Rad51 tolerate MMS-induced replicative DNA damage by cell cycle-regulated replicative and repair activities. The observation that the Mrc1-branch of the S-phase checkpoint prevents the assembly of MMS-induced Rad52 foci explains why stressed forks require HR yet at the same time activate a mechanism that inhibits HR: the DNA replication checkpoint inhibits the repair but not the replicative functions of Rad52 and Rad51 27 (Fig. 3). According to this model, Mrc1 would prevent aberrant Rad51-dependent recombination structures in response to hydroxyurea by inhibiting unscheduled repair activities of the recombination proteins traveling with the fork. It is worth noting that the temporal accumulation of MMS-induced HR foci in G2 is not lost in the absence of Mec1 or Rad53 27. This observation, together with the fact that Mec1 and Rad53, but not Mrc1, are required for the stability of stressed forks 90, rules out the possibility that the accumulation of HR foci in S phase in *mrc1* mutants is due to replication fork collapse.

While the recruitment of HR proteins to DSBs is independent of DNA replication 12,105, an important and distinctive feature of the HR response to alkylated DNA is that the binding of Rad52 and Rad51 to the forks during DNA replication is a prerequisite for the further repair of the ssDNA gaps. Thus, if the expression of yeast Rad52 is restricted to the G2 phase, Rad52 cannot bind to the ssDNA gaps, which remain unrepaired and do not form Rad52 foci 27. Rad52 and Rad51 most likely facilitate bypassing the blocking lesion and then remain bound to the ssDNA gaps left behind the fork until G2 (Fig. 3). The fact that the repair process requires Rad52 and Rad51 binding to the fork indicates that their replicative and repair activities are mechanistically connected although temporally separated.

Replication checkpoint activation by replicative stress inhibits the assembly of the HR centers during S phase 12,13,27. This function, whether or not it is associated with additional mechanisms, ensures that repair does not occur as long as there are stressed forks. Indeed, I think that the logic behind the DNA replication checkpoint-dependent inhibition of recombinational repair is linked to the fact that the repair occurs at HR centers. These centers are highly dynamic structures formed by giga-dalton-sized protein assemblies that are able to deal with multiple recombinogenic lesions, likely by concentrating recombination proteins and DNA substrates 100. This favorable environment for DSB repair might become a threat for genome integrity if the repair centers are assembled at stressed replication forks. Such a situation would interfere with proper DNA replication, which occurs at replication factories 108. In fact, it might promote fork restart by a mutagenic BIR process rather than by a safer canonical replisome. Further, the proximity of additional sequences at the repair centers could favor multiple rounds of homology and microhomology-driven BIR and FoSTeS events, which have been proposed to explain single cellular catastrophes (referred to as chromothripsis 109) that are associated with cancer and genetic diseases 54–57 (Fig. 3).

How the replication checkpoint inhibits the assembly of HR centers is currently unknown. The formation of repair centers in yeast is independent of Rad52 and therefore of any recombination intermediate subsequent to the accumulation of ssDNA 27. Since ssDNA fragments are already present at the lesions induced by DNA adducts and replication inhibitors, one possibility is that Mrc1 prevents ssDNA extension as a necessary step to form the repair centers. Consistent with this idea, Mrc1 activation by MMS inhibits DNA resection at DSBs 12. Additionally, the kinase activity of the yeast CDK Cdc28, which is required for DNA resection at DSBs 110,111, is necessary for the assembly of MMS-induced Rad52 foci 27. Therefore, the amount of either ssDNA or RPA might regulate the formation of the HR foci. In this context, it is tempting to speculate that the tumor suppressor genes BRCA1 and BRCA2, as well as other factors that control the amount of ssDNA/RPA, might protect against HR-mediated genetic instability by regulating the formation of repair centers.

Therefore, I think that a major task of the replicative checkpoints is to prevent the formation of HR repair centers under replicative stress as they can favor genome rearrangements. An important implication of this function is related to cancer progression. Oncogene-induced alterations in replication dynamics cause an accumulation of replicative stress by unknown mechanisms. This stress leads to checkpoint activation, which act as an early barrier to tumor progression by promoting senescence or preventing genetic instability through DNA repair/tolerance mechanisms. Consistently, mutations that impair this barrier facilitate tumor progression at later stages 1–3. According to my hypothesis, the assembly of HR centers during oncogene-induced replicative stress in checkpoint mutants would boost genetic instability.

A mechanistic weakness of this model is the fact that the DNA damage checkpoint, which is activated by DSBs, does not prevent the formation of HR centers despite it operates through the same effectors as the replication checkpoint. Mrc1 might inhibit the assembly of HR centers by different unknown effectors. However, and in contrast to Rad53 in *S. cerevisiae* 27, the *S. pombe* ortholog Cds1 is sufficient to prevent the formation of hydroxyurea-induced HR foci in S phase 13. Additionally, the mediator activity of Rad9 is able to inhibit DNA resection and HR foci formation in response to replicative stress in a *mrc1*^*AQ*^ checkpoint-defective mutant 12,27. A deeper insight to the many connections between the DNA damage and replication checkpoints in response to different stresses is still missing. In fact, the involvement of Cdc28 in HR foci formation 27 points to a much more complex response that also involves S and M cyclins.

## Conclusions

In summary, an important strategy to avoid genetic instability during the rescue of stressed replication forks by HR seems to be to inhibit HR repair centers by the Mrc1 branch of the S phase checkpoints. It is also likely that the DNA replication checkpoint prevents specific recombination intermediates from accumulating at the forks, and this may differ depending on the stress conditions. Once replication is over and the replication checkpoint is turned off, HR centers can be assembled to complete the repair of the accumulated ssDNA gaps and broken ends.

A number of important questions remain to be resolved. The analysis of BRCA2 and Rad51 mutations suggests that the stabilization of Rad51 filaments by BRCA2 is dispensable for DSB repair but required for protecting hydroxyurea-stressed forks from Mre11 degradation 24. The discovery of a similar dual role for HR in yeast provides a powerful genetic tool to search for additional separation-of-function mutations that help us to define the replicative and repair activities of HR. Additionally, as previously underlined, future experiments should focus on the relevance of DNA resection in the formation of HR centers. Indeed, we need to advance our understanding of the biology of HR centers as an essential part of the DNA damage response. Although model organisms such as yeast continue to be a helpful tool to approach these problems, parallel studies in higher eukaryotes are necessary for assessing the potential impact of the proposed model in the genetic instability associated with cancer and other genetic disorders. In particular, the regulatory role that Claspin plays in the regulation of DNA repair centers in response to replicative stress should be clarified. Understanding the mechanisms that ensure that the replicative and repair activities of HR occur in distinct subnuclear structures at different cell cycle stages may help us to understand how cells prevent an accumulation of genetic instability during DNA replication.

## Abbreviations

BIR: break-induced replication
CDK: cyclin-dependent kinase
DSB: double-strand break
FoSTeS: fork stalling and template switching
HJ: Holliday junction
HR: homologous recombination
ssDNA: single-stranded DNA
MMS: methyl-methane sulfonate
NHEJ: non-homologous end joining

## References

[1] Halazonetis TD, Gorgoulis VG, Bartek J (2008). An oncogene-induced DNA damage model for cancer development. Science.

[2] Gorgoulis VG, Vassiliou L-VF, Karakaidos P, Zacharatos P (2005). Activation of the DNA damage checkpoint and genomic instability in human precancerous lesions. Nature.

[3] Bartkova J, Horejsí Z, Koed K, Krämer A (2005). DNA damage response as a candidate anti-cancer barrier in early human tumorigenesis. Nature.

[4] Mirkin EV, Mirkin SM (2007). Replication fork stalling at natural impediments. Microbiol Mol Biol Rev.

[5] Helmrich A, Ballarino M, Nudler E, Tora L (2013). Transcription–replication encounters, consequences and genomic instability. Nat Struct Mol Biol.

[6] Boiteux S, Guillet M (2004). Abasic sites in DNA: repair and biological consequences in *Saccharomyces cerevisiae*. DNA Repair.

[7] Lambert S, Carr AM (2013). Impediments to replication fork movement: stabilization, reactivation and genome instability. Chromosoma.

[8] Poli J, Tsaponina O, Crabbé L, Keszthelyi A (2012). dNTP pools determine fork progression and origin usage under replication stress. EMBO J.

[9] Clemente-Ruiz M, Prado F (2009). Chromatin assembly controls replication fork stability. EMBO Rep.

[10] Clemente-Ruiz M, González-Prieto R, Prado F (2011). Histone H3K56 acetylation, CAF1, and Rtt106 coordinate nucleosome assembly and stability of advancing replication forks. PLoS Genet.

[11] Moynahan ME, Jasin M (2010). Mitotic homologous recombination maintains genomic stability and suppresses tumorigenesis. Nature.

[12] Alabert C, Bianco JN, Pasero P (2009). Differential regulation of homologous recombination at DNA breaks and replication forks by the Mrc1 branch of the S-phase checkpoint. EMBO J.

[13] Meister P, Taddei A, Vernis L, Poidevin M (2005). Temporal separation of replication and recombination requires the intra-S checkpoint. J Cell Biol.

[14] San Filippo J, Sung P, Klein H (2008). Mechanism of eukaryotic homologous recombination. Annu Rev Biochem.

[15] Marians KJ (2004). Mechanisms of replication fork restart in *Escherichia coli*. Philos Trans R Soc Lond B Biol Sci.

[16] Roseaulin L, Yamada Y, Tsutsui Y, Russell P (2008). Mus81 is essential for sister chromatid recombination at broken replication forks. EMBO J.

[17] Hashimoto Y, Puddu F, Costanzo V (2011). RAD51- and MRE11-dependent reassembly of uncoupled CMG helicase complex at collapsed replication forks. Nat Struct Mol Biol.

[18] Moriel-Carretero M, Aguilera A (2010). A postincision-deficient TFIIH causes replication fork breakage and uncovers alternative Rad51- or Pol32-mediated restart mechanisms. Mol Cell.

[19] Malkova A, Ira G (2013). Break-induced replication: functions and molecular mechanism. Curr Opin Genet Dev.

[20] Lydeard JR, Lipkin-Moore Z, Sheu YJ, Stillman B (2010). Break-induced replication requires all essential DNA replication factors except those specific for pre-RC assembly. Genes Dev.

[21] Lettier G, Feng Q, de Mayolo AA, Erdeniz N (2006). The role of DNA double-strand breaks in spontaneous homologous recombination in *S. cerevisiae*. PLoS Genet.

[22] Fabre F, Chan A, Heyer W-D, Gangloff S (2002). Alternate pathways involving Sgs1/Top3, Mus81/Mms4, and Srs2 prevent formation of toxic recombination intermediates from single-stranded gaps created by DNA replication. Proc Natl Acad Sci USA.

[23] Andersen PL, Xu F, Xiao W (2008). Eukaryotic DNA damage tolerance and translesion synthesis through covalent modifications of PCNA. Cell Res.

[24] Schlacher K, Christ N, Siaud N, Egashira A (2011). Double-strand break repair-independent role for BRCA2 in blocking stalled replication fork degradation by MRE11. Cell.

[25] Daboussi F, Courbet S, Benhamou S, Kannouche P (2008). A homologous recombination defect affects replication-fork progression in mammalian cells. J Cell Sci.

[26] Vázquez MV, Rojas V, Tercero JA (2008). Multiple pathways cooperate to facilitate DNA replication fork progression through alkylated DNA. DNA Repair.

[27] González-Prieto R, Muñoz-Cabello AM, Cabello-Lobato MJ, Prado F (2013). Rad51 replication fork recruitment is required for DNA damage tolerance. EMBO J.

[28] Lopes M, Foiani M, Sogo JM (2006). Multiple mechanisms control chromosome integrity after replication fork uncoupling and restart at irreparable UV lesions. Mol Cell.

[29] Hashimoto Y, Ray Chaudhuri A, Lopes M, Costanzo V (2010). Rad51 protects nascent DNA from Mre11-dependent degradation and promotes continuous DNA synthesis. Nat Struct Mol Biol.

[30] Liberi G, Maffioletti G, Lucca C, Chiolo I (2005). Rad51-dependent DNA structures accumulate at damaged replication forks in sgs1 mutants defective in the yeast ortholog of BLM RecQ helicase. Genes Dev.

[31] Courcelle J, Donaldson JR, Chow K-H, Courcelle CT (2003). DNA damage-induced replication fork regression and processing in *Escherichia coli*. Science.

[32] Yoon D, Wang Y, Stapleford K, Wiesmüller L (2004). p53 inhibits strand exchange and replication fork regression promoted by human Rad51. J Mol Biol.

[33] Cobb JA, Bjergbaek L (2006). RecQ helicases: lessons from model organisms. Nucleic Acids Res.

[34] Petermann E, Helleday T (2010). Pathways of mammalian replication fork restart. Nat Rev Mol Cell Biol.

[35] McIlwraith MJ, Vaisman A, Liu Y, Fanning E (2005). Human DNA polymerase η promotes DNA synthesis from strand invasion intermediates of homologous recombination. Mol Cell.

[36] Kannouche P (2001). Domain structure, localization, and function of DNA polymerase eta, defective in xeroderma pigmentosum variant cells. Genes Dev.

[37] Chow KH (2003). RecO acts with RecF and RecR to protect and maintain replication forks blocked by UV-induced DNA damage in *Escherichia coli*. J Biol Chem.

[38] Schwartz EK, Heyer W-D (2011). Processing of joint molecule intermediates by structure-selective endonucleases during homologous recombination in eukaryotes. Chromosoma.

[39] Petermann E, Orta ML, Issaeva N, Schultz N (2010). Hydroxyurea-stalled replication forks become progressively inactivated and require two different RAD51-mediated pathways for restart and repair. Mol Cell.

[40] López-Contreras AJ, Ruppen I, Nieto-Soler M, Murga M (2013). A proteomic characterization of factors enriched at nascent DNA molecules. Cell Reports.

[41] Sirbu BM, Couch FB, Feigerle JT, Bhaskara S (2011). Analysis of protein dynamics at active, stalled, and collapsed replication forks. Genes Dev.

[42] Bansbach CE, Betous R, Lovejoy CA, Glick GG (2009). The annealing helicase SMARCAL1 maintains genome integrity at stalled replication forks. Genes Dev.

[43] Hombauer H, Campbell CS, Smith CE, Desai A (2011). Visualization of eukaryotic DNA mismatch repair reveals distinct recognition and repair intermediates. Cell.

[44] Kliszczak AE, Rainey MD, Harhen B, Boisvert FM (2011). DNA mediated chromatin pull-down for the study of chromatin replication. Sci Rep.

[45] Mladenov E, Iliakis G (2011). Induction and repair of DNA double strand breaks: the increasing spectrum of non-homologous end joining pathways. Mutat Res.

[46] Waters LS, Minesinger BK, Wiltrout ME, D'Souza S (2009). Eukaryotic translesion polymerases and their roles and regulation in DNA damage tolerance. Microbiol Mol Biol Rev.

[47] Hicks WM, Kim M, Haber JE (2010). Increased mutagenesis and unique mutation signature associated with mitotic gene conversion. Science.

[48] Deem A, Keszthelyi A, Blackgrove T, Vayl A (2011). Break-induced replication is highly inaccurate. PLoS Biol.

[49] Donnianni RA, Symington LS (2013). Break-induced replication occurs by conservative DNA synthesis. Proc Natl Acad Sci USA.

[50] Wilson MA, Kwon Y, Xu Y, Chung W-H (2013). Pif1 helicase and Polδ promote recombination-coupled DNA synthesis via bubble migration. Nature.

[51] Saini N, Ramakrishnan S, Elango R, Ayyar S (2013). Migrating bubble during break-induced replication drives conservative DNA synthesis. Nature.

[52] Myung K, Datta A, Chen C, Kolodner RD (2001). *SGS1*, the *Saccharomyces cerevisiae* homologue of BLM and WRN, suppresses genome instability and homeologous recombination. Nat Genet.

[53] Wu L, Hickson ID (2003). The Bloom's syndrome helicase suppresses crossing over during homologous recombination. Nature.

[54] Lee JA, Carvalho CMB, Lupski JR (2007). A DNA replication mechanism for generating nonrecurrent rearrangements associated with genomic disorders. Cell.

[55] Hastings PJ, Ira G, Lupski JR (2009). A microhomology-mediated break-induced replication model for the origin of human copy number variation. PLoS Genet.

[56] Liu P, Erez A, Nagamani SCS, Dhar SU (2011). Chromosome catastrophes involve replication mechanisms generating complex genomic rearrangements. Cell.

[57] Zhang F, Khajavi M, Connolly AM, Towne CF (2009). The DNA replication FoSTeS/MMBIR mechanism can generate genomic, genic and exonic complex rearrangements in humans. Nature.

[58] Smith CE, Llorente B, Symington LS (2007). Template switching during break-induced replication. Nature.

[59] Payen C, Koszul R, Dujon B, Fischer G (2008). Segmental duplications arise from Pol32-dependent repair of broken forks through two alternative replication-based mechanisms. PLoS Genet.

[60] Bignell GR, Santarius T, Pole JCM, Butler AP (2007). Architectures of somatic genomic rearrangement in human cancer amplicons at sequence-level resolution. Genome Res.

[61] Kadyk LC, Hartwell LH (1992). Sister chromatids are preferred over homologs as substrates for recombinational repair in *Saccharomyces cerevisiae*. Genetics.

[62] González-Barrera S, Cortés-Ledesma F, Wellinger RE, Aguilera A (2003). Equal sister chromatid exchange is a major mechanism of double-strand break repair in yeast. Mol Cell.

[63] Prado F, Cortés-Ledesma F, Huertas P, Aguilera A (2003). Mitotic recombination in *Saccharomyces cerevisiae*. Curr Genet.

[64] Lambert S, Mizuno K, Blaisonneau J, Martineau S (2010). Homologous recombination restarts blocked replication forks at the expense of genome rearrangements by template exchange. Mol Cell.

[65] Harrison JC, Haber JE (2006). Surviving the breakup: the DNA damage checkpoint. Annu Rev Genet.

[66] Heyer W-D, Ehmsen KT, Liu J (2010). Regulation of homologous recombination in eukaryotes. Annu Rev Genet.

[67] Huertas P (2010). DNA resection in eukaryotes: deciding how to fix the break. Nat Struct Mol Biol.

[68] Lydeard JR, Lipkin-Moore Z, Jain S, Eapen VV (2010). Sgs1 and Exo1 redundantly inhibit break-induced replication and de novo telomere addition at broken chromosome ends. PLoS Genet.

[69] Mimitou EP, Symington LS (2010). Ku prevents Exo1 and Sgs1-dependent resection of DNA ends in the absence of a functional MRX complex or Sae2. EMBO J.

[70] Lengsfeld BM, Rattray AJ, Bhaskara V, Ghirlando R (2007). Sae2 is an endonuclease that processes hairpin DNA cooperatively with the Mre11/Rad50/Xrs2 complex. Mol Cell.

[71] Limbo O, Chahwan C, Yamada Y, de Bruin RAM (2007). Ctp1 is a cell-cycle-regulated protein that functions with Mre11 complex to control double-strand break repair by homologous recombination. Mol Cell.

[72] Sartori AA, Lukas C, Coates J, Mistrik M (2007). Human CtIP promotes DNA end resection. Nature.

[73] Gravel S, Chapman JR, Magill C, Jackson SP (2008). DNA helicases Sgs1 and BLM promote DNA double-strand break resection. Genes Dev.

[74] Mimitou EP, Symington LS (2008). Sae2, Exo1 and Sgs1 collaborate in DNA double-strand break processing. Nature.

[75] Zhu Z, Chung W-H, Shim EY, Lee SE (2008). Sgs1 helicase and two nucleases Dna2 and Exo1 resect DNA double-strand break ends. Cell.

[76] Chen X, Niu H, Chung W-H, Zhu Z (2011). Cell cycle regulation of DNA double-strand break end resection by Cdk1-dependent Dna2 phosphorylation. Nat Struct Mol Biol.

[77] Huertas P, Cortés-Ledesma F, Sartori AA, Aguilera A (2008). CDK targets Sae2 to control DNA-end resection and homologous recombination. Nature.

[78] Huertas P, Jackson SP (2009). Human CtIP mediates cell cycle control of DNA end resection and double strand break repair. J Biol Chem.

[79] Yun MH, Hiom K (2009). BRCA1 modulates the choice of DNA double- strand-break repair pathway throughout the cell cycle. Nature.

[80] Bunting SF, Callén E, Wong N, Chen H-T (2010). 53BP1 inhibits homologous recombination in Brca1-deficient cells by blocking resection of DNA breaks. Cell.

[81] Zou L (2007). Single- and double-stranded DNA: building a trigger of ATR-mediated DNA damage response. Genes Dev.

[82] Tercero JA, Longhese MP, Diffley JFX (2003). A central role for DNA replication forks in checkpoint activation and response. Mol Cell.

[83] Zou L (2013). Four pillars of the S-phase checkpoint. Genes Dev.

[84] Gilbert CS, Green CM, Lowndes NF (2001). Budding yeast Rad9 is an ATP-dependent Rad53 activating machine. Mol Cell.

[85] Lee J, Kumagai A, Dunphy WG (2003). Claspin, a Chk1-regulatory protein, monitors DNA replication on chromatin independently of RPA, ATR, and Rad17. Mol Cell.

[86] Alcasabas AA, Osborn AJ, Bachant J, Hu F (2001). Mrc1 transduces signals of DNA replication stress to activate Rad53. Nat Cell Biol.

[87] Tanaka K, Russell P (2001). Mrc1 channels the DNA replication arrest signal to checkpoint kinase Cds1. Nat Cell Biol.

[88] Mochan TA, Venere M, DiTullio RA, Halazonetis TD (2004). 53BP1, an activator of ATM in response to DNA damage. DNA Repair.

[89] Osborn AJ, Elledge SJ (2003). Mrc1 is a replication fork component whose phosphorylation in response to DNA replication stress activates Rad53. Genes Dev.

[90] Tourrière H, Versini G, Cordón-Preciado V, Alabert C (2005). Mrc1 and Tof1 promote replication fork progression and recovery independently of Rad53. Mol Cell.

[91] Katou Y, Kanoh Y, Bando M, Noguchi H (2003). S-phase checkpoint proteins Tof1 and Mrc1 form a stable replication–pausing complex. Nature.

[92] Tanaka K (2010). Multiple functions of the S-phase checkpoint mediator. Biosci Biotechnol Biochem.

[93] Branzei D, Foiani M (2009). The checkpoint response to replication stress. DNA Repair.

[94] Cotta-Ramusino C, Fachinetti D, Lucca C, Doksani Y (2005). Exo1 processes stalled replication forks and counteracts fork reversal in checkpoint-defective cells. Mol Cell.

[95] Forment JV, Blasius M, Guerini I, Jackson SP (2011). Structure-specific DNA endonuclease Mus81/Eme1 generates DNA damage caused by Chk1 inactivation. PLoS One.

[96] Kai M (2005). Replication checkpoint kinase Cds1 regulates Mus81 to preserve genome integrity during replication stress. Genes Dev.

[97] Betous R, Mason AC, Rambo RP, Bansbach CE (2012). SMARCAL1 catalyzes fork regression and Holliday junction migration to maintain genome stability during DNA replication. Genes Dev.

[98] Couch FB, Bansbach CE, Driscoll R, Luzwick JW (2013). ATR phosphorylates SMARCAL1 to prevent replication fork collapse. Genes Dev.

[99] Barlow JH, Rothstein R (2010). Timing is everything: cell cycle control of Rad52. Cell Div.

[100] Lisby M, Rothstein R (2004). DNA damage checkpoint and repair centers. Curr Opin Cell Biol.

[101] Lisby M, Antúnez de Mayolo A, Mortensen UH, Rothstein R (2003). Cell cycle-regulated centers of DNA double-strand break repair. Cell Cycle.

[102] Lisby M, Barlow JH, Burgess RC, Rothstein R (2004). Choreography of the DNA damage response. Cell.

[103] Aten JA, Stap J, Krawczyk PM, van Oven CH (2004). Dynamics of DNA double-strand breaks revealed by clustering of damaged chromosome domains. Science.

[104] Meister P (2003). Nuclear factories for signalling and repairing DNA double strand breaks in living fission yeast. Nucleic Acids Res.

[105] Barlow JH, Rothstein R (2009). Rad52 recruitment is DNA replication independent and regulated by Cdc28 and the Mec1 kinase. EMBO J.

[106] Su X, Bernal JA, Venkitaraman AR (2008). Cell-cycle coordination between DNA replication and recombination revealed by a vertebrate N-end rule degron-Rad51. Nat Struct Mol Biol.

[107] Feng Z, Zhang J (2012). A dual role of BRCA1 in two distinct homologous recombination mediated repair in response to replication arrest. Nucleic Acids Res.

[108] Kitamura E, Blow JJ, Tanaka TU (2006). Live-cell imaging reveals replication of individual replicons in eukaryotic replication factories. Cell.

[109] Stephens PJ, Greenman CD, Fu B, Yang F (2011). Massive genomic rearrangement acquired in a single catastrophic event during cancer development. Cell.

[110] Ira G, Pellicioli A, Balijja A, Wang X (2004). DNA end resection, homologous recombination and DNA damage checkpoint activation require CDK1. Nature.

[111] Aylon Y, Liefshitz B, Kupiec M (2004). The CDK regulates repair of double-strand breaks by homologous recombination during the cell cycle. EMBO J.

